# Division of neuromuscular compartments and localization of the center of the highest region of muscle spindles abundance in deep cervical muscles based on Sihler’s staining

**DOI:** 10.3389/fnana.2024.1340468

**Published:** 2024-05-22

**Authors:** Danli Wang, Peng Chen, Fangfang Jia, Meng Wang, Junxi Wu, Shengbo Yang

**Affiliations:** Department of Human Anatomy, Zunyi Medical University, Zunyi, China

**Keywords:** deep cervical muscle, Sihler’s staining, neuromuscular compartment, muscle spindle, target localization

## Abstract

**Purpose:**

The overall distribution pattern of intramuscular nerves and the regions with the highest spindle abundance in deep cervical muscles have not been revealed. This study aimed to reveal neuromuscular compartmentalization and localize the body surface position and depth of the center of the region of highest muscle spindle abundance (CRHMSA) in the deep cervical muscles.

**Methods:**

This study included 36 adult cadavers (57.7 ± 11.5 years). The curved line joining the lowest point of the jugular notch and chin tip was designated as the longitudinal reference line (line L), and the curved line connecting the lowest point of the jugular notch and acromion was designated as the horizontal reference line (line H). Modified Sihler’s staining, hematoxylin–eosin staining and computed tomography scanning were employed to determine the projection points (P) of the CRHMSAs on the anterior surfaces of the neck. The positions (P_H_ and P_L_) of point P projected onto the H and L lines, and the depth of each CRHMSA, and puncture angle were determined using the Syngo system.

**Results:**

The scalenus posterior and longus capitis muscles were divided into two neuromuscular compartments, while the scalenus anterior and longus colli muscles were divided into three neuromuscular compartments. The scalenus medius muscle can be divided into five neuromuscular compartments. The P_H_ of the CRHMSA of the scalenus muscles (anterior, medius, and posterior), and longus capitis and longus colli muscles, were located at 36.27, 39.18, 47.31, 35.67, and 42.71% of the H line, respectively. The P_L_ positions were at 26.53, 32.65, 32.73, 68.32, and 51.15% of the L line, respectively. The depths of the CRHMSAs were 2.47 cm, 2.96 cm, 2.99 cm, 3.93 cm, and 3.17 cm, respectively, and the puncture angles were 87.13°, 85.92°, 88.21°, 58.08°, and 77.75°, respectively.

**Conclusion:**

Present research suggests that the deep cervical muscles can be divided into neuromuscular compartments; we recommend the locations of these CRHMSA as the optimal target for administering botulinum toxin A injections to treat deep cervical muscle dystonia.

## 1 Introduction

The deep cervical muscles include the lateral [scalenus anterior (SA), scalenus medius (SM), and scalenus posterior (SP)] and medial groups [longus capitis (LCa), longus colli (LCo), rectus capitis anterior, and rectus capitis lateralis] (Rondot et al., 1991). The main function of these muscles is to contract one side to flex the neck to the same side. Acting together, they draw the neck forward. Neurogenic thoracic outlet syndrome (NTOS), characterized by spasms (Braun et al., 2006; Sanders et al., 2008) or muscle hypertrophy (Baltopoulos et al., 2008) in the SA and SM muscles, narrows the space between them, leading to compression of the brachial plexus and subclavian arteries (Jones et al., 2019; Gilcrease-Garcia et al., 2020). In clinical practice, intramuscular injection of botulinum toxin A (BTX-A) is often the preferred treatment (Torriani et al., 2010; Simpson et al., 2016). Increased muscle tone in the LCa and LCo muscles can cause cervical ptosis and “double chin” posture (Comella, 2008; Flowers et al., 2011; Finsterer et al., 2015), resulting in abnormal neck movement, difficulty maintaining a straight-ahead gaze while walking, and dysphagia (Comella, 2008). The use of BTX-A in these muscles has demonstrated therapeutic effects in clinical settings (Flowers et al., 2011; Allison and Odderson, 2016; Reichel et al., 2016; Farrell et al., 2020).

The site of BTX-A action is the motor endplate. Staining of the human muscle motor endplate has been limited to fresh specimens. Previous studies have reported that the intramuscular nerve dense region (INDR) location is consistent with the motor endplate band and can serve as an alternative target for BTX-A (Amirali et al., 2007; Phadke et al., 2013). Other studies have indicated that the muscle spindle, a type of stretch receptor, is not uniformly distributed within the muscle (Banks, 2006), with higher abundance in INDR and motor endplate bands (Yu et al., 2023). After many central nervous system diseases (such as stroke), due to the weakened inhibition of upper motor neurons on the spinal cord, leading to increased excitability of alpha motor neurons, increased sensitivity of Ia type fibers and increased activity of gamma motor fibers in the muscle spindle, the hyperactive stretching reflex can be achieved through the α-γ loop, thereby exacerbating muscle spasms (Phadke et al., 2013). Maximizing BTX-A to block the intrafusal muscle fibers while minimizing its impact on extrafusal muscle fibers can improve the efficacy and rehabilitation quality of BTX-A for treating muscle spasms (Phadke et al., 2013). Recent animal studies have confirmed that the center of the region of highest muscle spindle abundance (CRHMSA) in the INDR represents the optimal target for BTX-A to block muscle spasms (Yu et al., 2023).

In addition, the SA muscle resection or dissection has been used to treat NTOS and myogenic torticollis (Dong and Yu, 2013; Johansen, 2021). Myocutaneous flap transplantation from the LCa and LCo muscles has been used for repairing skull base defects, pharyngeal wall defects, soft palate reconstruction, cleft palate, and velopharyngeal insufficiency (Sader et al., 2001; Haku et al., 2008; Zhang et al., 2017; Gross et al., 2019). Studies have demonstrated that muscle fibers innervated by independent primary nerve branches within the muscle can function as sensory-motor sub-bodies or neuromuscular compartments. Whole-muscle transplantation carries the risk of losing donor site function, compared to neuromuscular compartment transplantation, which can simultaneously balance the functions of the donor and recipient site (Hu et al., 2023; Kurtys et al., 2023).

Based on the aforementioned clinical needs, this study aimed to reveal the distribution pattern of intramuscular nerves in deep cervical muscles and the division of neuromuscular compartments. Subsequently, hematoxylin and eosin (HE) staining was employed to count and compare the highest abundance of muscle spindles in INDR, leading to the designation of the CRHMSA as the optimal blocking target for BTX-A. The surface puncture position and depth of CRHMSA were determined using spiral computed tomography (CT). The research results can provide morphological guidance for deep cervical muscle transplantation and BTX-A injection target localization.

## 2 Materials and methods

### 2.1 Specimens and ethics

Thirty-six adult Chinese cadavers [18 males and 18 females, aged 35–75 (57.7 ± 11.5) years] with no history of neuromuscular disease or neck deformation were included. The causes of death of these donors were cancer, heart disease or accidents. Among them, 24 adult cadavers (12 males and 12 females) were fixed with formalin to study intramuscular nerve distribution and muscle spindle abundance, while 12 freshly frozen cadavers (six males and six females) were used to localize the CRHMSA. The collection and use of specimens were approved by the Ethics Committee of Zunyi Medical University (2022-1-003).

### 2.2 Gross anatomical observation and reference line design

The cadavers were placed in the supine position. A longitudinal incision extended from the chin tip to the lowest point of the jugular notch, and a transverse incision ran from the chin tip through the lower edge of the mandible to the mastoid process tip. Another transverse incision, from the lowest point of the jugular notch to the lower edge of the clavicle, continued to the acromion. The skin and subcutaneous tissue were then tightly flipped outward, forming a layer against the muscle surface. The sternoclavicular joint was detached first, followed by outward flipping to expose the complete lateral group of deep cervical muscles. This was followed by detachment from the mandibular joint. In front of the anterior intervertebral space, the mandible, along with structures such as the hyoid bone, larynx, thyroid gland, hyoid superior and inferior muscle groups, trachea, and esophagus, was flipped forward and downward to fully expose the medial group of deep cervical muscles, including the LCa and LCo muscles. This exposure allowed for the examination of muscle morphology, muscle fibers’ origin and insertion, nerve sources and muscle entry points, and the presence of blood vessels accompanying the nerves. To facilitate describing the relationship between the BTX-A blocking target in the deep cervical muscle and bony landmarks, as well as the medial and lateral relationships, a longitudinal reference line (L-line) was created to connect the lowest point of the jugular notch (point a) to the chin tip (point b), and a horizontal reference line (H-line) was established to connect the lowest point of the jugular notch to the acromion (point c).

### 2.3 Modified Sihler’s staining method for displaying intramuscular nerve dense region

Five deep cervical muscles (SA, SM, SP, LCa, LCo) were removed from 12 formalin-fixed cadavers (rectus capitis anterior and rectus capitis lateralis, located too deep with very small muscle mass, were excluded from this study because the diseases they cause were not described). The modified Sihler’s staining process (Wang et al., 2020) was employed, involving steps as follows: the muscles were macerated in 3% potassium hydroxide and 0.4% hydrogen peroxide solution for 3–4 weeks; decalcified in Sihler I solution (12 parts of 1% chloral trichlorohydrate, two parts of glacial acetic acid, and two parts of glycerol) for 4 weeks and in Sihler II solution (12 parts of 1% chloral hydrate, two parts of glycerol, and 1 part of Ehrlich’s hematoxylin solution) for 4 weeks. It is ideal to decolorize the Sihler I solution for 4–20 h as the muscle was light purple and the nerve branches were black. These were neutralized in 0.05% lithium carbonate solution for 3 h with continuous stirring. For transparency, glycerol gradients of 40, 60, 80, and 100% were performed for 1 week each. Subsequently, the distribution pattern of the intramuscular nerves was observed on the X-ray reading box, images were captured, and sketches were drawn. The INDR was drawn in a frame round using Adobe Photoshop CC2019 software (Adobe Company, United States). The INDR and the center of the INDR (CINDR) percentage positions relative to muscle length and width were measured using CAD software (Autodesk, United States).

### 2.4 He staining and calculation of muscle spindle abundance

Another twelve formalin fixed cadavers underwent dissection to expose deep cervical muscles. Based on Sihler’s staining results, the corresponding positions of each INDR were determined, and adjacent structures were observed. Anesthesia puncture needles were subsequently used under direct vision to simulate the puncture procedure (All anatomical and exposed structures were layer by layer restored to their normal positions without suturing, and puncture was performed from superficial to deep without obstruction, without damaging blood vessels, nerves, and submandibular glands, etc.). An analysis was conducted to assess the successful puncture of the INDR, the risk of damage to major cervical vessels, nerves, submandibular glands, thyroid, and other organs, and the risk of accidental entry into the respiratory tract and esophagus. The appropriate INDR for puncture and localization was determined through analysis of the simulation results. These identified INDRs were subdivided into three small parts: upper, middle, and lower. They were subsequently weighed, dehydrated, embedded in paraffin, and cross-sectioned continuously, with a slice thickness of 5 μm. The tissue underwent cutting, followed by H&E staining: Xylene dewaxing, gradient ethanol dewaxing, hematoxylin staining for 10 min, washing with distilled water, 1% hydrochloric acid alcohol for colour separation, tap water washing for 5 min, eosin staining for 2–5 min, gradient ethanol dehydration, xylene transparency, neutral gum sealing. Consecutive sections were then reconstructed under a microscope, and muscle spindles were counted. The predicted number of muscle spindles was calculated using the formula (Spn = 20.5 m_n_^0.49^) (Banks, 2006), where Spn represents the predicted number of muscle spindles and m_n_ represents the muscle weight. Subsequently, the actual count was divided by the predicted number to determine the muscle spindle abundance. The differences in muscle spindle abundance among the three parts of the INDR were compared. Additionally, the region with the highest muscle spindle abundance and the location of the CRHMSA were identified.

### 2.5 Spiral CT localization of the CRHMSA

Twelve freshly frozen cadavers were thawed and dissected to expose five deep cervical muscles in this study. Using the INDR position obtained from Sihler’s staining and the CRHMSA position determined by HE staining, a mixture of barium sulfate powder (Shandong Jiashuo radiation Protection Engineering Co., Ltd., China) and 801 glue (Wenzhou 801 Glue Corporation Ltd., Wenzhou, China, according to the proportion of 4 kg/L medical barium sulfate powder and 801 glue) was injected into the muscle to label the CRHMSA, using a syringe. A puncture needle was inserted into the CRHMSA in the direction of the simulated puncture, and suturing was performed layer by layer. Subsequently, scanning and 3D reconstruction were conducted using a 64-slice spiral CT scanner (Siemens, Germany) with settings at 120 KV, sheet thickness 1 mm, collimation 64 × 0.75 mm, pitch 1:1. The CRHMSA for the SA, SM, SP, LCa, and LCo muscles were designated as CRHMSA_1_, CRHMSA_2_, CRHMSA_3_, CRHMSA_4_, and CRHMSA_5_, respectively. The total lengths of the H and L reference lines were measured close to the skin using a Syngo system (Siemens).

The projection points (P) of the CRHMSAs on the anterior surfaces of the neck is denoted as point P, the intersection of a straight line passing through point P, which is perpendicular to the H line, with the H line is denoted as P_H_ (P_1H_-P_5H_). Similarly, the intersection of a horizontal line passing through point P and the L line is denoted as P_L_ (P_1L_-P_5L_). The distance from point a to point b is equal to the length of the L line. The distance from point a to point c is equal to the length of the H line. The distance between points a and P_H_ is denoted as H′ (H_1_–H_5_’), and the distance between points a and P_L_ is denoted as L’ (L_1_–L_5_’). Calculations were performed for H′/H × 100% and L’/L × 100% to determine the percentage position of point P on the body surface. Additionally, the sharp angle between the puncture needle and the skin was recorded as θ_1_, θ_2_, θ_3,_ θ_4,_ and θ_5_. Measurements were taken for the angle θ and the depth of the CRHMSA.

### 2.6 Statistical processing

All experimental data were analyzed using SPSS 18.0 software (IBM Corp., Armonk, NY, United States) and expressed as percentages (mean ± SD, %) to account for individual height and weight differences. The measured data were normally distributed. Therefore, a paired *t*-test was employed to compare data between the left and right sides, an independent sample *t*-test was used to compare data between males and females, and a one-way analysis of variance was used to compare muscle spindle abundance at different sites. Statistical significance was set at *α* = 0.05, with *p* < 0.05 considered statistically significant.

## 3 Results

### 3.1 Intramuscular nerve distribution pattern and intramuscular position of the CRHMSA

**Figure 1:**
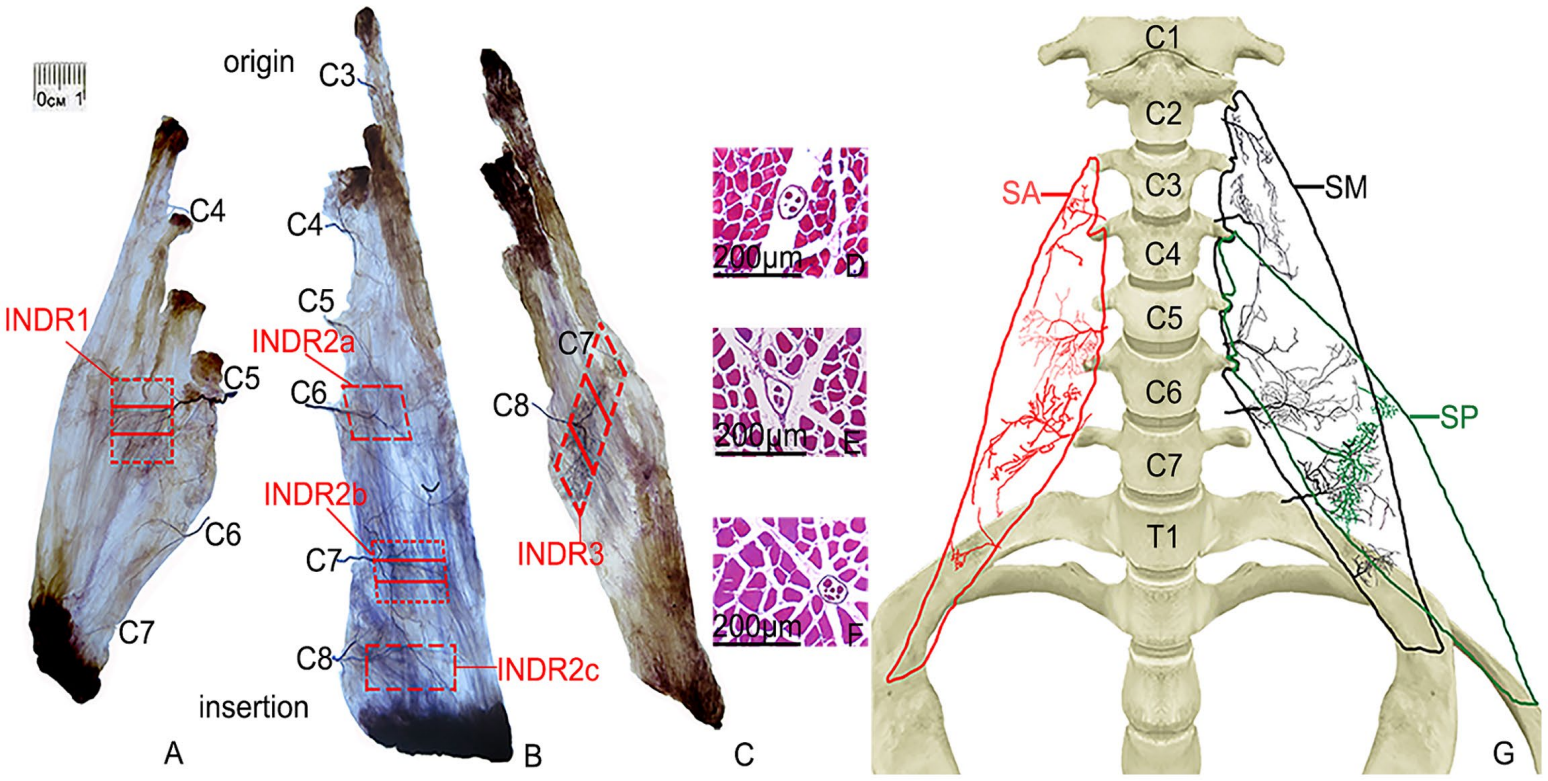
Sihler’s staining of the SA, SM, and SP muscles and representative muscle spindles in the highest region of muscle spindle abundance. **(A–C)** Sihler staining of the SA, SM, and SP muscles (superficial view). Red boxes indicate INDR1, INDR2a, INDR2b, INDR2c, and INDR3. **(D–F)** Representative muscle spindles with the highest abundance of INDR1, INDR2b, and INDR3 (scale: 200 μm). **(G)** Distribution pattern of the intramuscular nerves of the SA, SM, SP are shown in red, black, and green, respectively.

The SA muscle receives its nerve branch from C4 at the level of origin, which further divides into two primary branches: upper and lower branches. The upper branch, relatively small in size, follows a horizontal course outside, with its main branches distributed in the upper part of the muscle bundle originating from the third cervical transverse process. The lower branch extends outward and downward distributing to the upper muscle bundle originating from the transverse process of the third and fourth cervical vertebrae. As the nerve branch from C5 enters the middle of the muscle, it runs laterally and gradually emits more arborized branches distributed within the middle of the muscle belly. The arborized branches communicate with each other within the muscle, forming a square intramuscular nerve dense region (INDR1) with an area of approximately (1.55 ± 0.22) cm^2^ and located between (45.41 ± 0.32)% and (59.66 ± 0.40)% of the muscle length. After the nerve branch from C6 enters the lower part of muscle, it is divided into two primary branches, one extending superolaterally and the other inferolaterally, mainly innervating the lower part of the muscle bundle originating from the sixth cervical transverse process. Communication existed between the superolateral branch and the branch from C5. Occasionally, branches of C7 entered the muscle and were distributed in the muscle fibers on the medial side of the insertion. The muscle was divided into three neuromuscular compartments based on the independent innervation ranges of the C4, C5, and C6 nerve branches within the SA muscle. The highest region of muscle spindle abundance was located in the middle of INDR1, and its CRHMSA was located at the 51.87 ± 0.49% level of the muscle length (Figure 1).

The nerve branch of the SM muscle from C3 entered the muscle at the level of the first dentate origin, extending inferolaterally, with branches distributed within the muscle bundles originating from the second and third cervical vertebrae. After the nerve branch from C4 enters the muscle at the level of the third dentate origin, it bifurcates, with branches extending upward and downwards and distributed to the upper part of the muscle bundle, originating from the fourth cervical vertebra. Nerve branches from C5 run outwards and downwards, distributed in the upper part of the muscle bundle, originating from the fifth cervical vertebra. The nerve branches from C6 run horizontally outward in the sixth cervical vertebra, with many innervating the upper part of the muscle bundles of the fourth, fifth, and sixth cervical vertebrae and forming an intramuscular nerve dense region (INDR2a) through anastomosis with the branches of C5 in the middle of the muscle. The branches of C7 penetrate the lower part of muscle, emitting additional branches outward. In contrast, the branches from all levels anastomose in the middle and lower parts of the muscle to form an intramuscular nerve dense region (INDR2b), considered dense. After the C8 branches enter the muscle near the muscle insertion, they form arborized branches densely distributed in the lower part of the muscle, forming the intramuscular nerve dense region (INDR2c), which is regarded as the third. These three INDRs were located at (49.80 ± 0.22)% to (57.51 ± 0.29)%, (70.55 ± 0.21)% to (78.30 ± 0.23)%, and (84.51 ± 0.28) to (90.57 ± 0.23)% of the muscle’s length, with area of (0.95 ± 0.11) cm^2^, (0.92 ± 0.09) cm^2^, and (0.43 ± 0.04) cm^2^, respectively. Based on the independent innervation ranges of the C3, C4, C5-C6, C7, and C8 nerve branches within the muscle, the SM muscle was divided into five neuromuscular compartments. The region with the highest muscle spindle abundance was located in the middle of INDR2b, and its CRHMSA was located at approximately the (74.37 ± 0.90)% level of muscle length (Figure 1).

**Figure 2:**
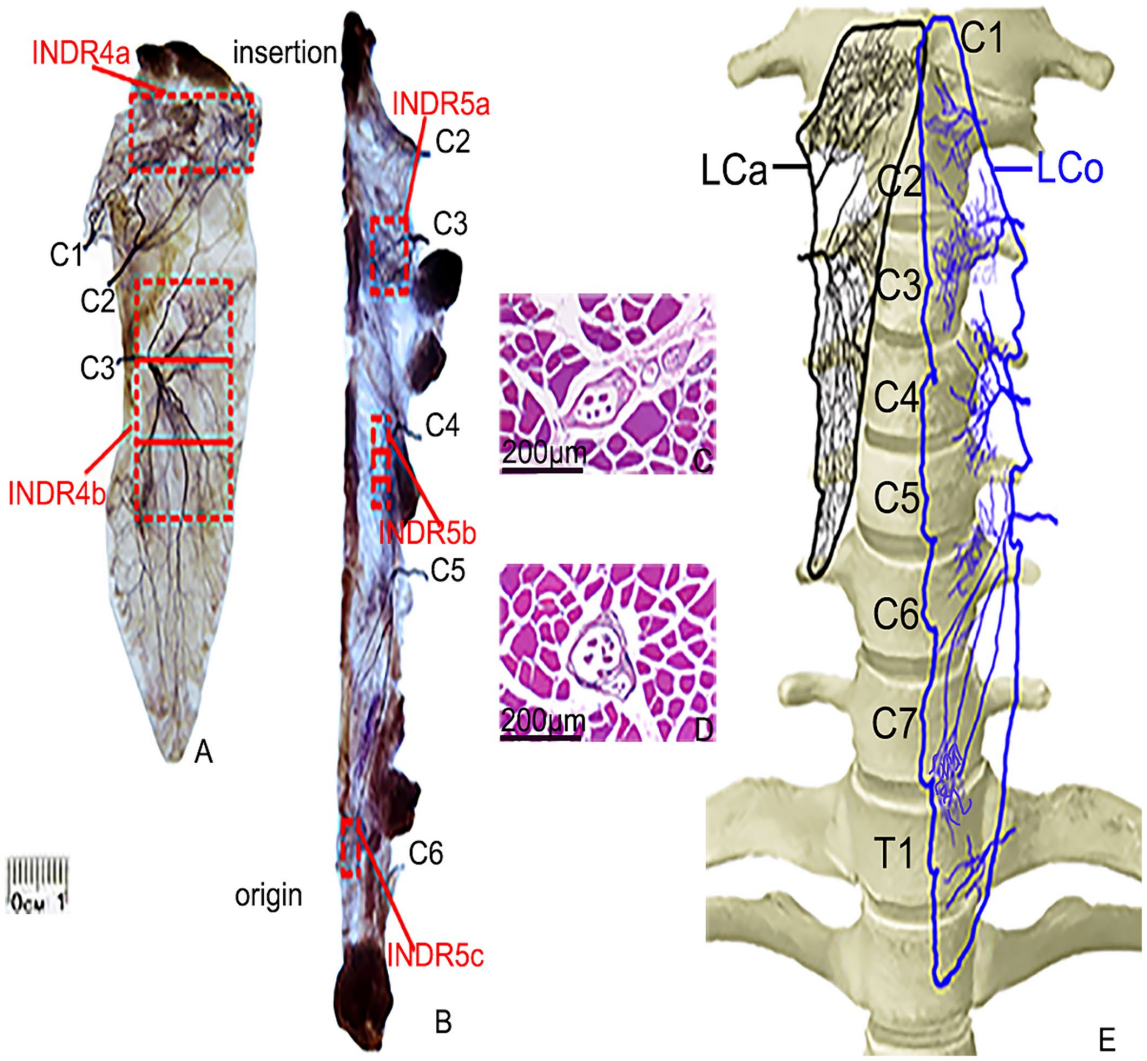
Sihler’s staining and representative muscle spindles in the highest region of muscle spindle abundance in the LCa and LCo muscles. **(A,B)** Sihler’s staining of the LCa and LCo (superficial view). Red boxes indicate INDR4a, INDR4b, INDR5a, INDR5b, and INDR5c. **(C,D)** Representative muscle spindles with the highest abundance in INDR4b and INDR5b (scale: 200 μm). **(E)** Distribution pattern of the intramuscular nerves of the LCa and LCo are shown in black and blue, respectively.

After the SP muscle nerve branch from C7 enters the upper part of muscle, the main dense branches are distributed in the middle of the muscle bundle, originating from the fourth cervical transverse process. When the nerve branch from C8 enters the middle part of muscle, the main dense branches are distributed in the middle of the muscle bundle, originating from the transverse processes of the fifth and sixth cervical vertebrae. These branches formed a band-shaped intramuscular nerve dense region (INDR3), located diagonally between (44.74 ± 0.14)% and (70.79 ± 0.24)% of the muscle length, covering an area of approximately (1.89 ± 0.08) cm^2^. Based on the independent innervation ranges of the C7 and C8 nerve branches within the muscle, the muscle was divided into two neuromuscular compartments. The region with the highest muscle spindle abundance was located in the middle of INDR3, with its CRHMSA located at approximately the (57.24 ± 0.67)% level of the muscle length (Figure 2).

After the LCa muscle nerve branches from C1 and C2 enter the upper part of muscle, they led to the discharge of 3–5 primary branches that ran diagonally inward and upwards until they reached the end of the muscle, following many branches which had extensive communication and jointly innervated the upper part of the muscle. INDR4a was formed at the level of (7.43 ± 0.13)% to (17.48 ± 0.19)% of muscle length. The nerve branches from C3 radiated gradually after entering the middle part of muscle, and there was communication between the nerve branches that ran inward and upward and the branches of C2. The nerve branches that ran inwards and downward were distributed throughout the lower part of the muscle. These dense arborized branches formed another intramuscular nerve dense region (INDR4b) at the level of (30.96 ± 0.20)% to (64.71 ± 0.27)% of muscle length. The area sizes of the two INDRs were (1.99 ± 0.18) cm^2^ and (4.5 ± 0.38) cm^2^, respectively. Based on the relatively independent innervation range of the C1, C2, and C3 nerve branches within the muscle, the muscle was divided into C1-C2 and C3 neuromuscular compartments. The region with the highest muscle spindle abundance was located in the middle of INDR4b, and its CRHMSA was located at the (47.28 ± 0.51)% level of muscle length (Figure 1).

After the LCo muscle nerve branches from C2 and C3 enter the muscle at the level of the corresponding number of muscle teeth, the branches are mainly distributed in the upper part of the muscle, especially in the C3 nerve branch, with more branches forming INDR5a through anastomosis. The branches of the C4 nerve extend medially upon entering the middle part of muscle and anastomose in the middle, forming a second intramuscular nerve dense region (INDR5b). When the C5 nerve branch enters the lower part of muscle, it often emits three primary branches that run inward and downward, with their branches anastomosing with each other in the lower part of the muscle, forming INDR5c. These three INDRs were located at the levels of (20.95 ± 0.14)% to (27.92 ± 0.23)%, (39.97 ± 0.19)% to (48.90 ± 0.14)%, and (78.95 ± 0.20)% to (83.70 ± 0.19)% of muscle length, respectively. The three INDRs areas are (0.74 ± 0.08) cm^2^, (0.55 ± 0.05) cm^2^, and (0.30 ± 0.03) cm^2^, respectively. The muscle exhibited relatively independent innervation ranges from the C2 and C3 nerve branches, as well as C4 and possibly C5, resulting in its subdivision into three neuromuscular compartments: C2-C3, C4, and C5. Additionally, limited C6 nerve innervation was observed at the muscle termination. The highest region of muscle spindle abundance was located in the middle of INDR5b, with its CRHMSA located at the (44.75 ± 0.38)% level of muscle length (Figure 2).

### 3.2 Muscle spindle abundance of INDR for puncture and localization

According to Sihler’s staining results, during the simulated puncture of each INDR on the cadaver, INDR1 of the SA muscle was not affected by its position; the position of INDR2a and INDR1 in the SM muscle overlapped, and there was a brachial plexus covering the upper part, while INDR2b was not affected by the adjacent structures. The risk of pleural cavity penetration is high when puncturing the INDR2c; the positions of the SP muscle’s INDR are not influenced; those of the LCa muscle and LCo muscle have an incorrect position (INDR4a) and INDR5a (increased depth), which can lead to damage of the submandibular gland. The INDR5c that punctures the LCo muscle, can penetrate the pleural cavity, posing the risk of damaging the lung apex. Therefore, it is recommended that INDR1, INDR2b, INDR3, INDR4b, and INDR5b be used as target regions for BTX-A injection. The abundance of muscle spindles in these five INDRs that are conducive to facilitate puncture and localization was the highest in the LCa muscle, followed by the INDR4b, INDR5b, INDR3, INDR2b, and INDR1. Among the three parts of the five INDRs conducive to puncture and localization, the middle part had the highest muscle spindle abundance, with a statistically significant difference (*p* < 0.05) (Table 1). The representative muscle spindles in the region of highest muscle spindles abundance were shown in Figures 1D–F, 2C,D. Their CRHMSAs were located at the levels of (51.87 ± 0.49)%, (74.37 ± 0.90)%, (57.24 ± 0.67)%, (47.28 ± 0.51)%, and (44.75 ± 0.38)% of muscle length, respectively.

**Table 1:** Comparison of muscle spindle abundance between different parts of INDR that facilitate puncture and localization in the deep cervical muscle (*n* = 24 sides, mean ± SD).

INDRs	INDR parts	Muscle weight (g)	Actual no.	Predicting no.	Relative abundance
INDR1	Upper one-third	0.38 ± 0.10	22.59 ± 1.24	12.32 ± 1.65	1.87 ± 0.28^*^
	Middle one-third	0.28 ± 0.06	21.38 ± 1.43	10.94 ± 1.05	1.98 ± 0.29
	Lower one-third	0.35 ± 0.05	22.23 ± 1.40	12.20 ± 0.86	1.83 ± 0.15^*^
INDR2b	Upper one-third	0.36 ± 0.06	23.11 ± 1.30	12.37 ± 0.96	1.88 ± 0.20^Δ^
	Middle one-third	0.32 ± 0.05	23.37 ± 1.60	11.75 ± 0.92	2.00 ± 0.21
	Lower one-third	0.31 ± 0.06	20.81 ± 1.31	11.47 ± 1.16	1.83 ± 0.20^Δ^
INDR3	Upper one-third	0.28 ± 0.06	21.01 ± 1.14	10.88 ± 1.12	1.95 ± 0.23^※^
	Middle one-third	0.28 ± 0.04	22.24 ± 1.43	10.91 ± 0.73	2.05 ± 0.18
	Lower one-third	0.32 ± 0.03	19.77 ± 0.80	11.79 ± 0.47	1.68 ± 0.08^※^
INDR4b	Upper one-third	0.48 ± 0.04	27.10 ± 1.57	14.33 ± 0.54	1.89 ± 0.12^▲^
	Middle one-third	0.43 ± 0.05	29.87 ± 1.27	13.59 ± 0.72	2.20 ± 0.14
	Lower one-third	0.46 ± 0.06	25.84 ± 0.90	14.04 ± 0.94	1.85 ± 0.15^▲^
INDR5b	Upper one-third	0.19 ± 0.04	16.93 ± 0.85	9.06 ± 1.04	1.90 ± 0.26^▼^
	Middle one-third	0.13 ± 0.02	15.87 ± 1.42	7.57 ± 0.55	2.11 ± 0.24
	Lower one-third	0.14 ± 0.02	15.33 ± 0.69	8.54 ± 0.46	1.80 ± 0.12^▼^

INDR1 = intramuscular nerve dense region in the SA muscle. INDR2 = intramuscular nerve dense region in the middle and lower parts of the SM muscle. INDR3: intramuscular nerve dense region in the SP muscle. INDR4b = intramuscular nerve dense region in the middle part in the LCa muscle. INDR5b: intramuscular nerve dense region in the middle part in the LCo muscle. ^*Δ※▲▼^ Compare with the middle part of INDR1, INDR2b, INDR3, INDR4b, and INDR5b, respectively, *p* < 0.05.

### 3.3 Simulated puncture determination of CRHMSA needle entry path under gross anatomy

**Figure 3:**
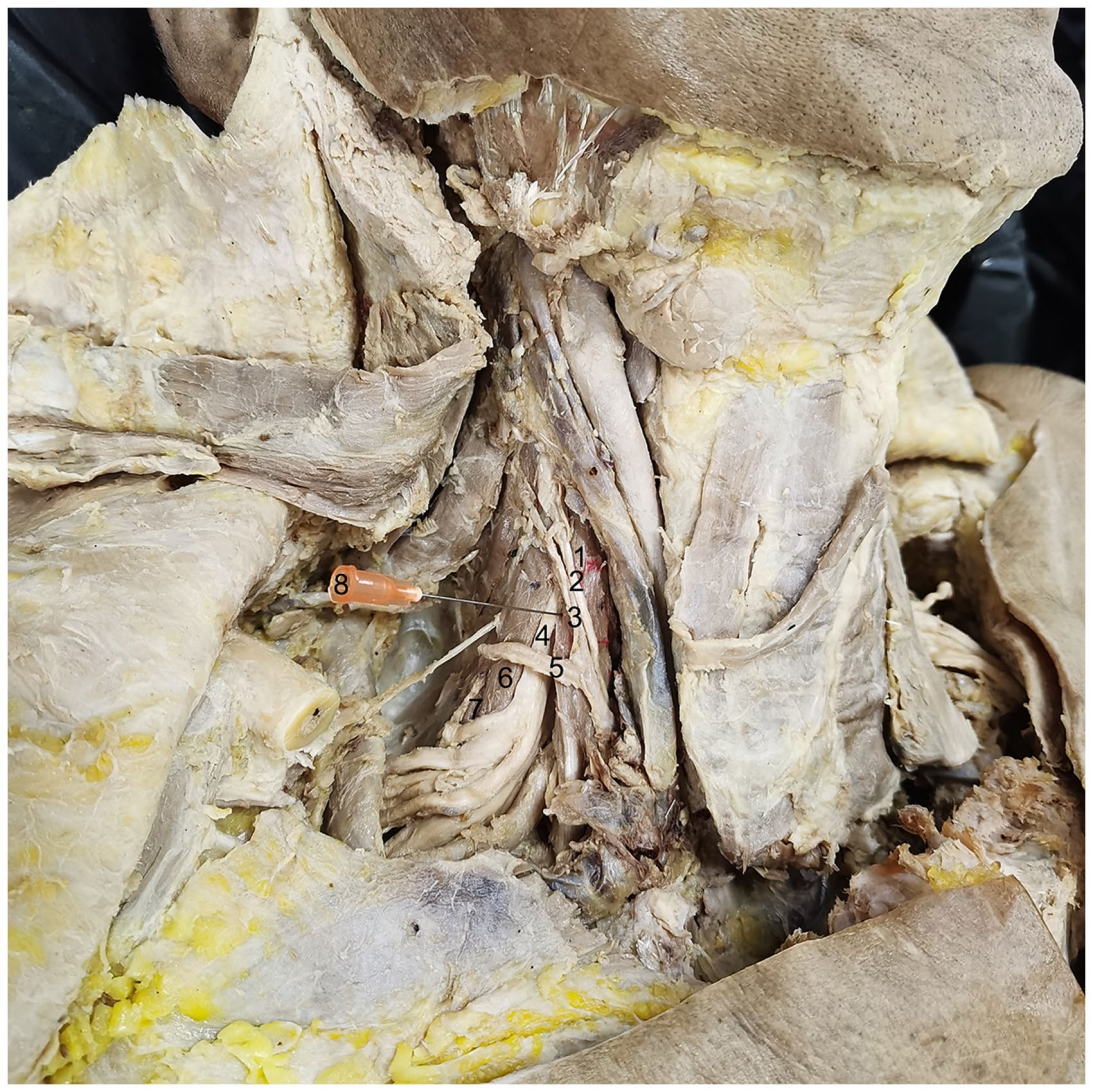
Simulated puncture of right SA muscle CRHMSA. 1: SA muscle, 2: phrenic nerve, 3: ascending cervical artery, 4: 5th cervical nerve (C5), 5: transverse cervical artery, 6: SM muscle, 7: SP muscle, 8: needle.

According to the CRHMSA location determined after HE staining, after the dissection and exposure of the deep cervical muscles, simulated puncture of the CRHMSA were performed. It was found that the insertion path for the lateral group of deep cervical muscles could be performed at a level two horizontal fingers above the clavicle. For the SA muscle, insertion was made from the lateral to the medial side, penetrating the skin, subcutaneous fat, platysma, and sternocleidomastoid muscle, ultimately reaching the space between the phrenic nerve and the lateral side of the ascending cervical artery, located above the transverse cervical artery. The SM muscle was inserted from the anterosuperior to the posteroinferior, passing through the skin, subcutaneous fat, platysma, and sternocleidomastoid muscle and reaching the space below the transverse cervical artery and lateral side of C5. The SP muscle was stabbed from the anterosuperior to the posteroinferior, passing through the skin, subcutaneous fat, latissimus muscle, and sternocleidomastoid muscle, finally reaching the angle formed by the suprascapular artery and the transverse cervical artery. The insertion path of the medial group of the deep neck muscle can be carried out at the hyoid level. The LCa muscle was punctured from to the anteroinferior to the posterosuperior, passing through the skin, subcutaneous fat, and platysma muscle to reach the space between the lateral side of the hyoid bone and the common carotid artery. In contrast, the LCo muscle required a puncture perpendicular to the skin at the level of the hyoid bone, passing through the skin, subcutaneous fat, and platysma muscle, and reaching through the space between the lateral side of the hyoid bone and the common carotid artery. This article presents the simulated puncture path of the right SA muscle of the CRHMSA (Figure 3), with specific positions and angles of needle insertion on the body surface listed in Table 2.

**Table 2:** Percentage position of CRHMSA on the body surface reference line, depth, and puncture angle (*n* = 24 sides, mean ± SD).

CRHMSA	P_H_ on the H line (%) H′/H (%)	P_L_ on the L line (%) L’/L (%)	Depth of CRHMSA (cm) P-CRHMSA	Puncture angle θ (°)
CRHMSA_1_	36.27 ± 1.65	26.53 ± 0.75	2.47 ± 0.07	87.13 ± 2.19
CRHMSA_2_	39.18 ± 1.93	32.65 ± 0.62	2.96 ± 0.19	85.92 ± 2.08
CRHMSA_3_	47.31 ± 1.13	32.73 ± 0.73	2.99 ± 0.21	88.21 ± 1.93
CRHMSA_4_	35.67 ± 1.64	68.32 ± 1.03	3.93 ± 0.07	58.08 ± 1.79
CRHMSA_5_	42.71 ± 1.09	51.15 ± 0.91	3.17 ± 0.16	77.75 ± 2.49

CRHMSA1 = center of the region of highest muscle spindle abundance of SA muscle spindle; CRHMSA2 = center of the region of highest muscle spindle abundance of SM muscle spindle; CRHMSA3 = center of the region of highest muscle spindle abundance of SP muscle spindle; CRHMSA4 = center of the region of highest muscle spindle abundance of LCa muscle spindle; CRHMSA5 = center of the region of highest muscle spindle abundance of LCo muscle spindle.

### 3.4 Spiral CT localization of the CRHMSA

**Figure 4:**
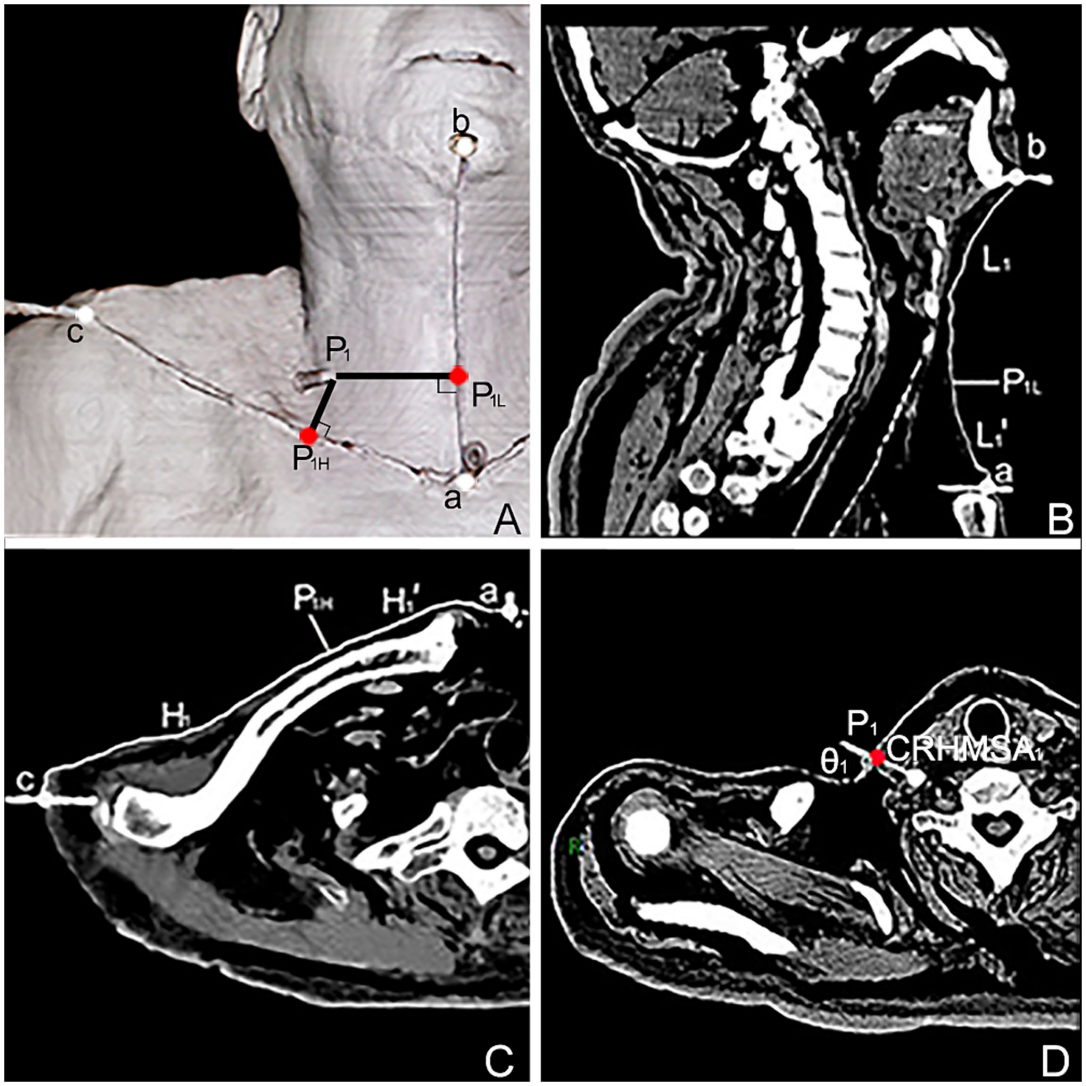
Spiral CT localization image of the center of the highest region of muscle spindle abundance (CRHMSA1) of the right SA muscle in males. **(A)** Three-dimensional reconstructed spiral CT image showing the position of CRHMSA1 on the body surface and the designed reference line. P1 is the surface projection point of the SA muscle CRHMSA1. P1H = intersection point generated by creating a line perpendicular to line H through P1. P1L = intersection point of the H-line passing through P1 and the L-line. a-P1H=H1’, a-P1L = L1’. **(B)** Lengths of the L and L1 lines in the coronal plane were measured. **(C)** Measure the lengths of the H and H1’ lines on the section passing through line H. **(D)** Determine the depth and angle of needle insertion for CRHMSA1 in the cross section.

The surface projection point (point P) of the CRHMSA projected the percentage position on the reference line, along with the angle between the puncture needle and the skin at the P point and the depth of the CRHMSA, as shown in Table 2. No statistically significant differences were observed between the left and right sides or between males and females (*p* > 0.05) (Tables 3–5). This article includes a spiral CT localization image of the CRHMSA, using the right SA muscle as an example (Figure 4).

**Table 3:** Comparison of the percentile position and depth of CRHMSA on the body surface reference line between males and females (*n* = 6, mean ± SD).

CRHMSA	P_H_ on the H line (%) H′/H (%)				P_L_ on the L line (%) L’/L (%)				Depth of CRHMSA (cm) P-CRHMSA			
	Males	Females	*t*	*p*	Males	Females	*t*	*p*	Males	Females	*t*	*p*
CRHMSA_1_	36.61 ± 1.54	35.93 ± 1.81	0.705	0.608	26.60 ± 0.95	26.47 ± 0.62	0.273	0.092	2.49 ± 0.07	2.45 ± 0.07	0.893	0.896
CRHMSA_2_	39.77 ± 2.33	38.60 ± 1.45	1.041	0.085	32.86 ± 0.58	32.45 ± 0.63	1.161	0.953	2.97 ± 0.18	2.95 ± 0.21	0.252	0.658
CRHMSA_3_	47.62 ± 1.13	47.00 ± 1.14	0.952	0.901	32.90 ± 0.70	32.63 ± 0.75	0.660	0.851	3.03 ± 0.19	2.95 ± 0.24	0.638	0.605
CRHMSA_4_	35.61 ± 1.83	35.74 ± 1.69	−0.126	0.864	68.33 ± 1.15	68.10 ± 1.01	0.365	0.708	3.96 ± 0.07	3.91 ± 0.07	1.224	0.927
CRHMSA_5_	42.89 ± 1.37	42.53 ± 0.88	0.539	0.499	51.05 ± 1.02	51.26 ± 0.91	−0.368	0.670	3.22 ± 0.17	3.12 ± 0.14	1.163	0.478

**Table 4:** Comparison of percentile positions and depths of left and right CRHMSA on the body surface reference line (*n* = 12, mean ± SD).

CRHMSA	P_H_ on the H line (%) H′/H (%)				P_L_ on the L line (%) L’/L (%)				Depth of CRHMSA (cm) P-CRHMSA			
	Left side	Right side	*t*	*P*	Left side	Right side	*t*	*P*	Left side	Right side	*t*	*P*
CRHMSA_1_	36.08 ± 1.47	36.46 ± 1.93	−1.327	0.211	26.52 ± 0.80	26.55 ± 0.74	−0.644	0.533	2.47 ± 0.07	2.48 ± 0.07	−1.149	0.275
CRHMSA_2_	39.11 ± 2.00	39.26 ± 1.94	−0.975	0.351	32.66 ± 0.58	32.64 ± 0.68	0.252	0.806	2.95 ± 0.21	2.97 ± 0.17	−0.835	0.422
CRHMSA_3_	47.34 ± 1.11	47.29 ± 1.19	0.344	0.737	32.77 ± 0.68	32.76 ± 0.74	0.153	0.881	2.99 ± 0.20	2.99 ± 0.23	0.118	0.908
CRHMSA_4_	35.66 ± 1.76	35.69 ± 1.60	−0.338	0.742	68.20 ± 0.98	68.21 ± 1.12	−0.070	0.945	3.95 ± 0.06	3.92 ± 0.08	1.732	0.111
CRHMSA_5_	42.72 ± 1.14	42.70 ± 1.10	0.354	0.730	51.11 ± 0.97	51.19 ± 0.90	−1.054	0.314	3.15 ± 0.15	3.18 ± 0.17	−1.707	0.116

**Table 5:** Comparison of puncture angles of CRHMSA between males and females and between left and right sides (mean ± SD).

CRHMSA	The angle between the puncture needle and the skin (°)							
	Males (*n* = 6)	Females (*n* = 6)	*t*	*P*	Males (*n* = 6)	Females (*n* = 6)	*t*	*p*
CRHMSA_1_	86.58 ± 2.46	87.67 ± 1.66	−0.894	0.140	87.42 ± 2.31	86.83 ± 2.12	1.292	0.223
CRHMSA_2_	85.83 ± 2.52	86.00 ± 1.52	−0.139	0.425	86.25 ± 1.82	85.58 ± 2.35	1.685	0.120
CRHMSA_3_	89.08 ± 1.32	87.33 ± 1.91	1.843	0.387	88.33 ± 1.61	88.08 ± 2.27	0.561	0.586
CRHMSA_4_	58.42 ± 1.91	57.75 ± 1.67	0.645	0.461	57.92 ± 1.68	58.25 ± 1.96	−1.076	0.305
CRHMSA_5_	79.00 ± 1.61	76.50 ± 2.59	2.008	0.313	77.67 ± 2.61	77.83 ± 2.48	−0.394	0.701

## 4 Discussion

As described in previous research, the SA muscle was innervated by C4–C7; the SM muscle by C2–C8, the SP muscle by C6–C8, the LCa muscle by C1–C3, and the LCo muscle by C2–C6 (Sakamoto, 2012). The results of this study are consistent with these findings. However, in this study, neither gross anatomical observations nor intramuscular nerve staining revealed the C2 nerve branch innervations of the SM muscle and the C6 nerve branch innervated the SP muscle. This discrepancy might be attributed to the relatively small sample size. Currently, no reports are available regarding the distribution pattern of intramuscular nerves in the deep cervical muscles. For the first time, this study employed the modified Sihler’s staining method to clearly display the overall distribution pattern of intramuscular nerves in the deep cervical muscle, which is visible to the naked eye. This method revealed the division of compartments within these muscles: the SP and LCa can be divided into two neuromuscular compartments; the SA and LCo can be divided into three neuromuscular compartments; the SM can be divided into five neuromuscular compartments. It is recommended to divide the LCa muscle flap into two parts when using it for the repair and reconstruction of the palatopharyngeal sphincter (Zhang et al., 2017), soft palate (Gross et al., 2019), or palatopharyngeal coloboma (Sader et al., 2001). At the origin of the third cervical vertebra, the upper muscle belly should be split diagonally inward to create a LCa muscle flap with a cranial pedicle, allowing for the safe harvesting of nutrient-rich muscle flaps (Sader et al., 2001; Zhang et al., 2017; Gross et al., 2019); when using the LCo muscle flap transplantation to repair esophageal perforation after cervical vertebrae surgery (Haku et al., 2008), it is recommended to select the matching neuromuscular compartment from among the three available compartments based on the required area size. Furthermore, if surgical resection of the scalene muscles is needed to reduce muscle tone for the treatment of NTOS (Johansen, 2021), it is recommended to cut or release the C5 compartment of the SA muscle, the C5-C6 and C7 compartments of the SM muscle, and the C8 compartment of the SP muscle, as these compartments have a dense nerve distribution.

Regarding the study of muscle spindles in deep cervical muscles, Boyd-Clark (Boyd-Clark et al., 2002) found that the average spindle density in the LCo muscle was 48.6/g, often concentrated in clusters of 2–10 in the anterolateral aspect of the muscle, and this density did not change with age. This study focused on muscle spindle abundance rather than muscle spindle density. No information is available on other deep cervical muscles, and there is no reported relationship between muscle spindle abundance and intramuscular nerve distribution. One study reported muscle spindles abundance of 1.8 in the SA muscle and 1.9 in the SM and SP muscles (Banks, 2006). The muscle spindles abundances obtained in this experiment were slightly higher than that in the mentioned study, possibly because of the uneven distribution of muscle spindles. This study focused on the INDR, which has a relatively large number of muscle spindles (Yu et al., 2023). Present results indicate that the CRHMSA in the deep cervical muscle aligns with the center of the INDR. In fact, the locations of the five CRHMSA revealed in this study are the center positions of the five INDR (INDR4b, INDR5b, INDR3, INDR2b, and INDR1) that are conducive to puncture and localization.

BTX-A is injected into the motor endplate to block the release of acetylcholine from the presynaptic membrane, thereby inhibiting muscle excitation and alleviating muscle spasms (Isner-Horobeti et al., 2016). Simultaneously, it alleviates nerve compression caused by muscle hypertrophy through denervation and muscle atrophy (Multani et al., 2019). Therefore, as mentioned in this article, the application of BTX-A injection for treating NTOS and other deep cervical muscle tone is very suitable. However, the effectiveness and safety of BTX-A depends on the proximity of the injection site to the motor endplate. When BTX-A was injected 5 mm from the effective target, its efficacy decreased by 50% (Shaari and Sanders, 1993). However, if the dosage was too high, it could produce antibodies, cause muscle fibrosis, increase the patient’s economic burden (Van Campenhout et al., 2013, 2015), and cause drug diffusion, leading to non-target muscle paralysis. Difficulty in swallowing and breathing were the most common side effects (Castagna and Albanese, 2019). In addition, BTX injection is skill driven in muscle selection and device-aided localization, which is also worth emphasizing. Based on the recommended infiltration of 1.5–3 cm^2^ per unit of BTX-A and 4.5 cm^2^ per 2.5–5 units (Borodic et al., 1994), combined with the recommended INDR area for puncture localization in this study, 1, 0.6, 1, 2.5 and 0.4 units can be injected into the CRHMSA of the SA, SM, SP, LCa, and LCo muscles, respectively. The need for additional doses can be determined based on their efficacy.

It is worth noting that the deep cervical muscles are located deep within the body, have relatively small muscle masses, and are surrounded by complex adjacent structures. It is recommended to perform procedures under ultrasound guidance. The CRHMSA of the SA muscle was located below the phrenic nerve and ascending cervical artery, and above C5. Therefore, it is necessary to insert needles in the gap between the two to avoid blood vessels and nerves. The upper part of the CRHMSA of the SM muscle was covered by the brachial plexus; therefore, the needle could not be inserted vertically from the front. As the transverse cervical artery passes through the lateral middle part of the SM muscle, and the brachial plexus nerve runs laterally through the scalene fissure, the needle can be inserted diagonally medial-inferior in the space between the transverse cervical artery and C5. The position of the CRHMSA in the SP muscle is more lateral and lower than that in the SM muscle. When inserting the needle diagonally downwards, it is important to avoid the lateral suprascapular artery. The anterior position of the CRHMSA of LCa muscle is covered by the trachea and esophagus, and the lateral side is adjacent to the carotid artery. The needle can be directed towards the cranial, and the needle moves diagonally between the inferolateral edge of the submandibular gland and the medial of the common carotid artery. The upper part of the LCo muscle CRHMSA is also covered by the trachea and esophagus, and the thyroid cartilage can be used as a landmark for needle insertion between the lateral edge of the thyroid cartilage plate and the medial of the common carotid artery.

In summary, this study systematically revealed the overall distribution pattern of intramuscular nerves in the deep cervical muscle for the first time. It explored the division of neuromuscular compartments and provided morphological guidance for the selection and design of surgical compartmentalized muscle transplantation to repair skull base defects, pharyngeal lateral wall defects, and soft palate reconstruction. In addition to the potential application of cutting off the scalene for the treatment of NTOS. Based on the higher abundance of muscle spindles in the deep cervical muscles compared to the superficial cervical muscles, the deep cervical muscles are the more impactful postural muscles, this study revealed the abundance of muscle spindle in the deep cervical muscle INDR and explored the CRHMSA in INDR that is conducive to puncture and localization. The study located the surface puncture position, determined the puncture depth, and measured the needle insertion angle, which can provide the best guidance for the localization of blocking targets for BTX-A injection treatment of deep cervical muscle tone enhancing diseases. However, this study did not involve racial differences, the sample size was relatively small, and the results were not validated for clinical applications.

## 5 Conclusion

Present research suggests that the deep cervical muscles can be divided into neuromuscular compartments; we recommend the locations of these CRHMSA as the optimal target for administering botulinum toxin A injections to treat deep cervical muscle dystonia.

## Data availability statement

The original contributions presented in the study are included in the article/supplementary material, further inquiries can be directed to the corresponding author.

## Ethics statement

The studies involving humans were approved by Ethics Committee of Zunyi Medical University (2022-1-003). The studies were conducted in accordance with the local legislation and institutional requirements. The participants provided their written informed consent to participate in this study. Written informed consent was obtained from the individual(s) for the publication of any potentially identifiable images or data included in this article.

## Author contributions

DW: Conceptualization, Data curation, Formal analysis, Investigation, Methodology, Writing – original draft. PC: Data curation, Formal analysis, Investigation, Methodology, Writing – original draft. FJ: Data curation, Formal analysis, Investigation, Methodology, Writing – original draft. MW: Data curation, Formal analysis, Investigation, Methodology, Writing – original draft. JW: Data curation, Formal analysis, Investigation, Writing – original draft. SY: Conceptualization, Funding acquisition, Writing – review & editing.

## References

[ref1] Allison S. K. Odderson I. R. (2016). Ultrasound and electromyography guidance for injection of the longus Colli with botulinum toxin for the treatment of cervical dystonia. Ultrasound Q. 32, 302–306. doi: 10.1097/RUQ.0000000000000226, PMID: 26886108

[ref2] Amirali A. Mu L. Gracies J. M. Simpson D. M. (2007). Anatomical localization of motor endplate bands in the human biceps brachii. J. Clin. Neuromuscul. Dis. 9, 306–312. doi: 10.1097/CND.0b013e31815c13a7, PMID: 18090684

[ref3] Baltopoulos P. Tsintzos C. Prionas G. Tsironi M. (2008). Exercise-induced scalenus syndrome. Am. J. Sports Med. 36, 369–374. doi: 10.1177/0363546507312166, PMID: 18202297

[ref4] Banks R. W. (2006). An allometric analysis of the number of muscle spindles in mammalian skeletal muscles. J. Anat. 208, 753–768. doi: 10.1111/j.1469-7580.2006.00558.x, PMID: 16761976 PMC2100235

[ref5] Borodic G. E. Ferrante R. Pearce L. B. Smith K. (1994). Histologic assessment of dose-related diffusion and muscle fiber response after therapeutic botulinum a toxin injections. Mov. Disord. 9, 31–39. doi: 10.1002/mds.870090106, PMID: 8139603

[ref001] Boyd-Clark L. C. Briggs C. A. Galea M. P. (2002). Muscle spindle distribution, morphology, and density in longus colli and multifidus muscles of the cervical spine. Spine (Phila Pa 1976). 27, 964–701. doi: 10.1097/00007632-200204010-00005, PMID: 11923661

[ref6] Braun R. M. Sahadevan D. C. Feinstein J. (2006). Confirmatory needle placement technique for scalene muscle block in the diagnosis of thoracic outlet syndrome. Tech. Hand Up. Extrem. Surg. 10, 173–176. doi: 10.1097/01.bth.0000231967.74041.48, PMID: 16974223

[ref7] Castagna A. Albanese A. (2019). Management of cervical dystonia with botulinum neurotoxins and EMG/ultrasound guidance. Neurol Clin Pract. 9, 64–73. doi: 10.1212/CPJ.0000000000000568, PMID: 30859009 PMC6382379

[ref8] Comella C. L. (2008). The treatment of cervical dystonia with botulinum toxins. J. Neural Transm. (Vienna) 115, 579–583. doi: 10.1007/s00702-007-0831-4 17994181

[ref9] Dong X. Yu D. (2013). Application of cicatricial contracture release principles in muscular torticollis treatment. Aesth. Plast. Surg. 37, 950–955. doi: 10.1007/s00266-013-0122-4, PMID: 23949124

[ref10] Farrell M. Karp B. I. Kassavetis P. Berrigan W. Yonter S. Ehrlich D.. (2020). Management of Anterocapitis and Anterocollis: a novel ultrasound guided approach combined with electromyography for botulinum toxin injection of longus Colli and longus capitis. Toxins (Basel) 12:626. doi: 10.3390/toxins12100626, PMID: 33008043 PMC7650774

[ref11] Finsterer J. Maeztu C. Revuelta G. J. Reichel G. Truong D. (2015). Collum-caput (COL-CAP) conceptfor conceptual anterocollis, anterocaput, and forward sagittal shift. J. Neurol. Sci. 355, 37–43. doi: 10.1016/j.jns.2015.06.015, PMID: 26088286

[ref12] Flowers J. M. Hicklin L. A. Marion M. H. (2011). Anterior and posterior sagittal shift in cervical dystonia: a clinical and electromyographic study, including a new EMG approach of the longus colli muscle. Mov. Disord. 26, 2409–2414. doi: 10.1002/mds.23905, PMID: 21913223

[ref13] Gilcrease-Garcia B. M. Deshmukh S. D. Parsons M. S. (2020). Anatomy, imaging, and pathologic conditions of the brachial plexus. Radiographics 40, 1686–1714. doi: 10.1148/rg.2020200012, PMID: 33001787

[ref14] Gross J. H. Zenga J. Sharon J. D. Jackson R. S. Pipkorn P. (2019). Longus capitis reconstruction of the soft palate. Otolaryngol. Head Neck Surg. 161, 536–538. doi: 10.1177/0194599819849031, PMID: 31084255

[ref15] Haku T. Okuda S. Kanematsu F. Oda T. Miyauchi A. Yamamoto T.. (2008). Repair of cervical esophageal perforation using longus colli muscle flap: a case report of a patient with cervical spinal cord injury. Spine J. 8, 831–835. doi: 10.1016/j.spinee.2007.06.017, PMID: 18082458

[ref16] Hu X. Wang M. He X. Chen P. Jia F. Wang D.. (2023). Division of neuromuscular compartments and localization of the center of the intramuscular nerve-dense region in pelvic wall muscles based on Sihler's staining. Anat. Sci. Int. 99, 127–137. doi: 10.1007/s12565-023-00744-4, PMID: 37768515 PMC10771363

[ref17] Isner-Horobeti M. E. Muff G. Lonsdorfer-Wolf E. Deffinis C. Masat J. Favret F.. (2016). Use of botulinum toxin type a in symptomatic accessory soleus muscle: first five cases. Scand. J. Med. Sci. Sports 26, 1373–1378. doi: 10.1111/sms.12616, PMID: 26627136

[ref18] Johansen K. (2021). Rib-sparing scalenectomy for neurogenic thoracic outlet syndrome: early results. J. Vasc. Surg. 73, 2059–2063. doi: 10.1016/j.jvs.2020.12.052, PMID: 33340695

[ref19] Jones M. R. Prabhakar A. Viswanath O. Urits I. Green J. B. Kendrick J. B.. (2019). Thoracic outlet syndrome: a comprehensive review of pathophysiology, diagnosis, and treatment. Pain Ther. 8, 5–18. doi: 10.1007/s40122-019-0124-2 31037504 PMC6514035

[ref20] Kurtys K. Podgórski M. Gonera B. Vazquez T. Olewnik Ł. (2023). An assessment of the variation of the intramuscular innervation of the gracilis muscle, with the aim of determining its neuromuscular compartments. J. Anat. 242, 354–361. doi: 10.1111/joa.13785, PMID: 36308488 PMC9919504

[ref21] Multani I. Manji J. Tang M. J. Herzog W. Howard J. J. Graham H. K. (2019). Sarcopenia, cerebral palsy and botulinum toxin type A. JBJS Rev 7:e4. doi: 10.2106/JBJS.RVW.18.00153, PMID: 31415277

[ref22] Phadke C. P. On A. Y. Kirazli Y. Ismail F. Boulias C. (2013). Intrafusal effects of botulinum toxin injections for spasticity: revisiting a previous paper. Neurosci. Lett. 541, 20–23. doi: 10.1016/j.neulet.2013.02.025, PMID: 23458671

[ref23] Reichel G. Stenner A. von Sanden H. Herrmann L. Feja C. Löffler S. (2016). Endoscopic-guided injection of botulinum toxin into the longus capitis muscle and into the obliquus superior part of the longus colli muscle in dystonic antecaput: our experience. Basal Ganglia 6, 97–100. doi: 10.1016/j.baga.2016.01.007

[ref24] Rondot P. Marchand M. P. Dellatolas G. (1991). Spasmodic torticollis--review of 220 patients. Can. J. Neurol. Sci. 18, 143–151. doi: 10.1017/S0317167100031619, PMID: 2070297

[ref25] Sader R. Zeilhofer H. F. Putz R. Horch H. H. (2001). Levatorplasty, a new technique to treat hypernasality: anatomical investigations and preliminary clinical results. J. Craniomaxillofac. Surg. 29, 143–149. doi: 10.1054/jcms.2001.0215, PMID: 11465252

[ref26] Sakamoto Y. (2012). Spatial relationships between the morphologies and innervations of the scalene and anterior vertebral muscles. Ann. Anat. 194, 381–388. doi: 10.1016/j.aanat.2011.11.004, PMID: 22209543

[ref27] Sanders R. J. Hammond S. L. Rao N. M. (2008). Thoracic outlet syndrome: a review. Neurologist 14, 365–373. doi: 10.1097/NRL.0b013e318176b98d 19008742

[ref28] Shaari C. M. Sanders I. (1993). Quantifying how location and dose of botulinum toxin injections affect muscle paralysis. Muscle Nerve 16, 964–969. doi: 10.1002/mus.880160913, PMID: 8355728

[ref29] Simpson D. M. Hallett M. Ashman E. J. Comella C. L. Green M. W. Gronseth G. S.. (2016). Practice guideline update summary: botulinum neurotoxin for the treatment of blepharospasm, cervical dystonia, adult spasticity, and headache: report of the guideline development Subcommittee of the American Academy of neurology. Neurology 86, 1818–1826. doi: 10.1212/WNL.0000000000002560, PMID: 27164716 PMC4862245

[ref30] Torriani M. Gupta R. Donahue D. M. (2010). Botulinum toxin injection in neurogenic thoracic outlet syndrome: results and experience using a ultrasound-guided approach. Skeletal Radiol. 39, 973–980. doi: 10.1007/s00256-010-0897-1, PMID: 20186413

[ref31] Van Campenhout A. Bar-On L. Desloovere K. Molenaers G. (2015). Role of motor end plate-targeted botulinum toxin type a injections in children with cerebral palsyitle. Acta Orthop. Belg. 81, 167–171, PMID: 26280952

[ref32] Van Campenhout A. Verhaegen A. Pans S. Molenaers G. (2013). Botulinum toxin type a injections in the psoas muscle of children with cerebral palsy: muscle atrophy after motor end plate-targeted injections. Res. Dev. Disabil. 34, 1052–1058. doi: 10.1016/j.ridd.2012.11.016, PMID: 23295965

[ref33] Wang J. Wang Q. Zhu D. Jiang Y. Yang S. (2020). Localization of the center of the intramuscular nerve dense region of the medial femoral muscles and the significance for blocking spasticity. Ann. Anat. 231:151529. doi: 10.1016/j.aanat.2020.151529 32437866

[ref34] Yu J. Li Y. Yang L. Li Y. Zhang S. Yang S. (2023). The highest region of muscle spindle abundance should be the optimal target of botulinum toxin a injection to block muscle spasms in rats. Front. Neurol. 14:1061849. doi: 10.3389/fneur.2023.1061849, PMID: 36908586 PMC9996071

[ref35] Zhang X. Y. Ma T. T. Liu L. Yin N. B. Zhao Z. M. (2017). Anatomic study of the musculus longus capitis flap. Surg. Radiol. Anat. 39, 271–279. doi: 10.1007/s00276-016-1708-8, PMID: 27289229

