# Supplementary figures and images for: Division of neuromuscular compartments and localization of the center of the highest region of muscle spindles abundance in deep cervical muscles based on Sihler’s staining
Source: Front Neuroanat. 2024 May 22;18:1340468. doi: 10.3389/fnana.2024.1340468 (PMC11151460; doi:10.3389/fnana.2024.1340468)

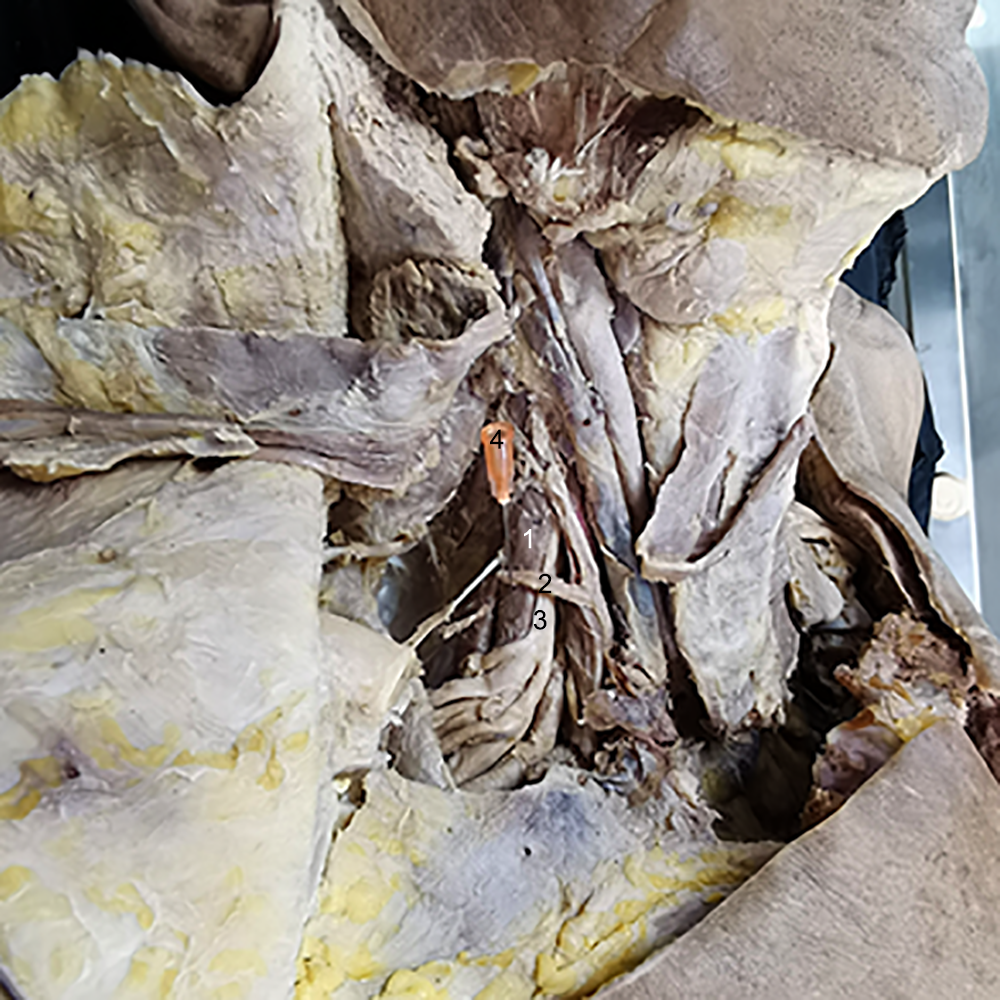

Supplement: Supplementary file 1 [file Image_1.TIF]

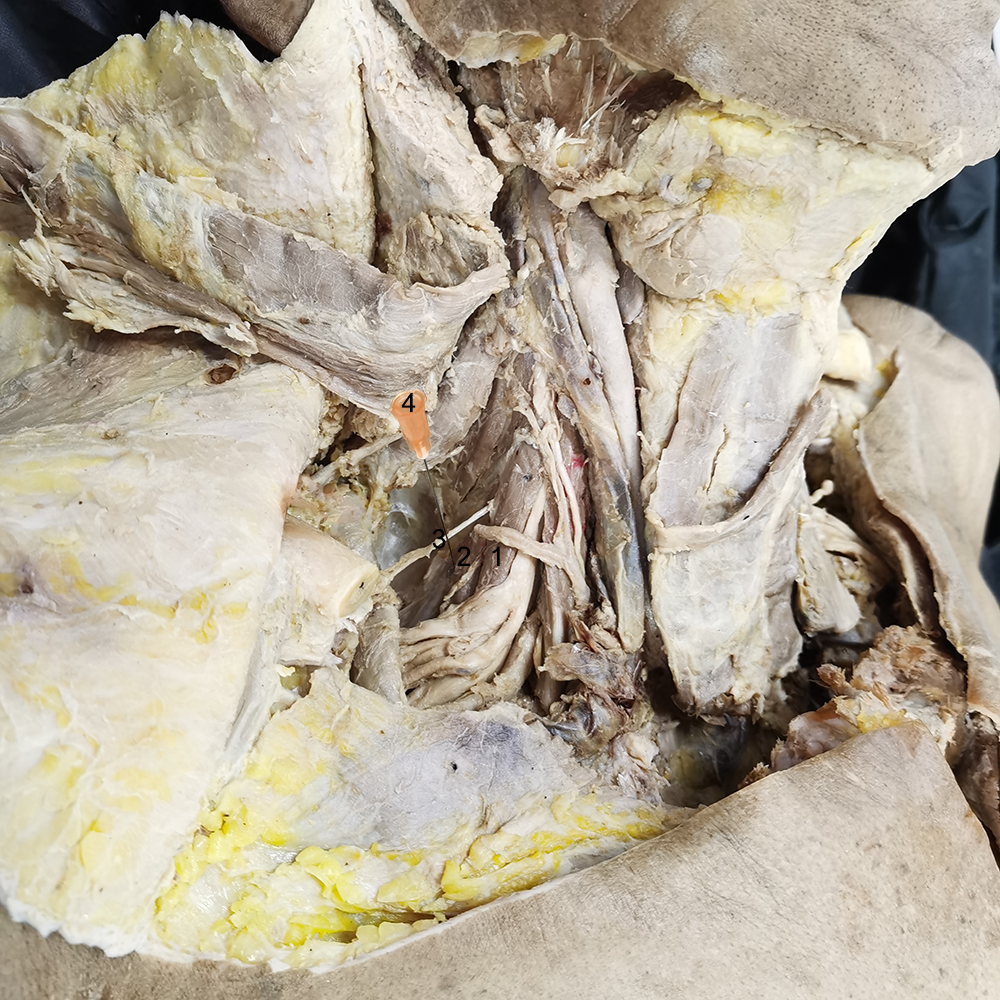

Supplement: Supplementary file 2 [file Image_2.TIF]

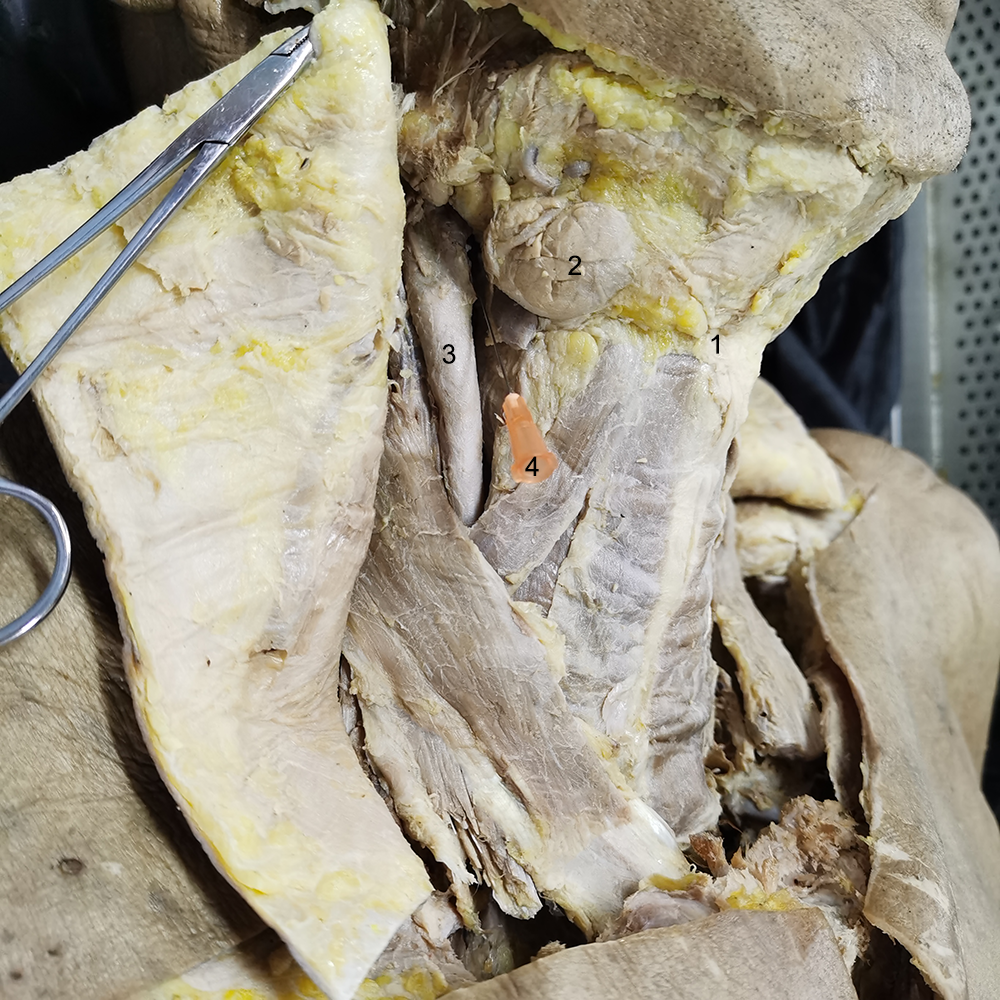

Supplement: Supplementary file 3 [file Image_3.TIF]

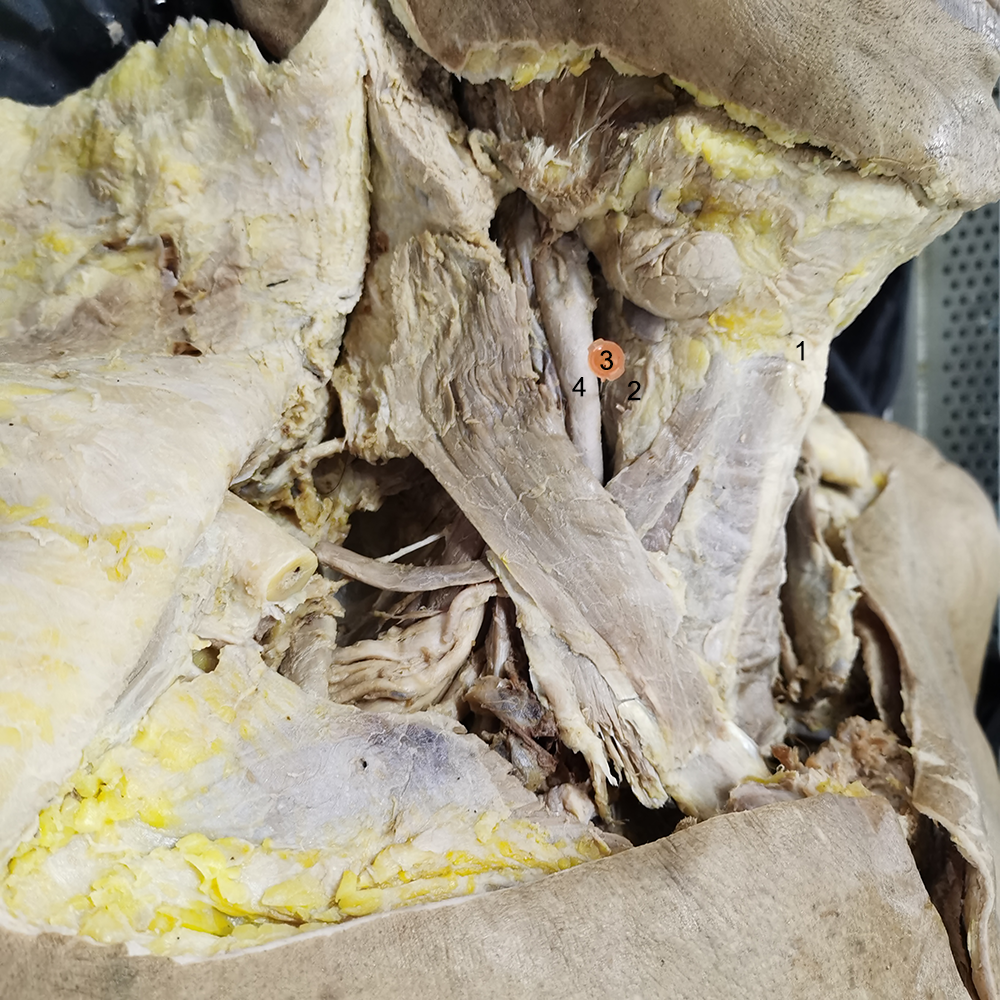

Supplement: Supplementary file 4 [file Image_4.TIF]
